# *LTC-Mapping*, Enhancing Long-Term Consistency of Object-Oriented Semantic Maps in Robotics

**DOI:** 10.3390/s22145308

**Published:** 2022-07-15

**Authors:** Jose-Luis Matez-Bandera, David Fernandez-Chaves, Jose-Raul Ruiz-Sarmiento, Javier Monroy, Nicolai Petkov, Javier Gonzalez-Jimenez

**Affiliations:** 1 Machine Perception and Intelligent Robotics Group (MAPIR-UMA), Malaga Institute for Mechatronics Engineering and Cyber-Physical Systems (IMECH.UMA), University of Malaga, 29016 Malaga, Spain; josematez@uma.es (J.-L.M.-B.); davfercha@uma.es (D.F.-C.); jotaraul@uma.es (J.-R.R.-S.); javiergonzalez@uma.es (J.G.-J.); 2 Johann Bernoulli Institute of Mathematics and Computing Science, University of Groningen, 9712 CP Groningen, The Netherlands; n.petkov@rug.nl

**Keywords:** semantic maps, object-oriented maps, long-term consistency, instance duplication, dynamic scenes, mobile robots, object detection, Detectron2

## Abstract

This paper proposes *LTC-Mapping*, a method for building object-oriented semantic maps that remain consistent in the long-term operation of mobile robots. Among the different challenges that compromise this aim, *LTC-Mapping* focuses on two of the more relevant ones: preventing duplicate instances of objects (instance duplication) and handling dynamic scenes. The former refers to creating multiple instances of the same physical object in the map, usually as a consequence of partial views or occlusions. The latter deals with the typical assumption made by object-oriented mapping methods that the world is static, resulting in outdated representations when the objects change their positions. To face these issues, we model the detected objects with 3D bounding boxes, and analyze the visibility of their vertices to detect occlusions and partial views. Besides this geometric modeling, the boxes are augmented with semantic information regarding the categories of the objects they represent. Both the geometric entities (bounding boxes) and their semantic content are propagated over time through data association and a fusion technique. In addition, in order to keep the map curated, the non-detection of objects in the areas where they should appear is also considered, proposing a mechanism that removes them from the map once there is evidence that they have been moved (i.e., multiple non-detections occur). To validate our proposal, a number of experiments have been carried out using the Robot@VirtualHome ecosystem, comparing its performance with a state-of-the-art alternative. The results report a superior performance of *LTC-Mapping* when modeling both geometric and semantic information of objects, and also support its online execution.

## 1. Introduction

In the context of mobile robotics, object-oriented semantic mapping refers to the process of building and maintaining a reliable representation of the objects found in the robot workspace by linking their geometric information (e.g., pose, size, shape, etc.) with their semantics (e.g., object types, functionalities, events, relations, etc.) [1,2,3]. An example of the latter may be that televisions are devices, usually placed in living rooms that can entertain people and are operated by a remote controller. In this context, semantic maps are crucial for an efficient robot operation, since they provide the robot with the level of understanding of the elements in human-centered environments required to operate with them [4,5]. For example, if the user tells the robot “*Hey! I’m bored*”, the robot could react by turning on the TV. Some typical robotic tasks that could benefit from the exploitation of these maps could be object search [6], navigation [7] or human–robot interaction [8], among others [3,9,10].

**Figure 1:**
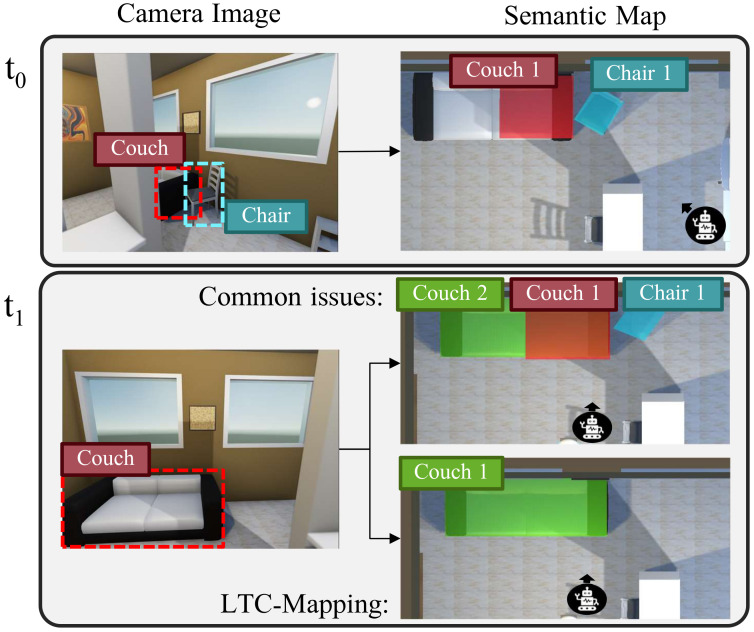
Illustration of the effects of two main challenges that compromise the reliability of semantic maps: instance duplication and dynamic objects. For each time moment *t*, we show the results yielded by the object detector (**left**) and the updated semantic map (**right**). At time $t_{0}$, a chair and a partially visible couch are detected and instantiated in the map. However, at time $t_{1}$, the previously detected chair has been moved and the couch is entirely visible. Ignoring the aforementioned challenges leads to the semantic map referred as Common issues, where two different instances refer to the same physical couch and the instance of the chair remains.

While object-oriented semantic maps bring clear benefits to intelligent robots, two main challenges appear to keep them effective for long-term robot operation: avoiding duplicated object instantiations and coping with dynamic objects [11,12,13]. Let us clarify these concepts. On the one hand, there is a need to check whether a new recognized object corresponds to an already instantiated object in the map (then, it must be updated), or to a new entity not seen before, in which case it must be incorporated in the map. Since object detection is usually performed from images where the objects may be partially visible—either due to occlusions from other objects or because it does not fall entirely within the camera field-of-view—this association task becomes cumbersome. Failing to associate partial observations of the same physical object seriously compromises the reliability of the resulting map, leading to the instance duplication problem, i.e., a single real object is instantiated multiple times (see Figure 1). On the other hand, there is another non-negligible issue that could seriously hamper the reliability of object-oriented semantic maps over time: the assumption that the world is static [11]. This simplification is inconsistent with the reality of human-centered environments (e.g., a house or an office), where most objects are highly likely to be moved. For example, in Figure 1, under this assumption, the representation of Chair 1 remains in the map even though the chair is no longer there. This fact turns the resulting map into a time-stamped snapshot, which does not truthfully represent the real-world.

In this paper, we present *LTC-Mapping*, a method for the construction and maintenance of object-oriented semantic maps from RGB-D images which specifically addresses the aforementioned challenges, i.e., instance duplication and dynamic objects. Concretely, an off-the-shelf object detector is used to detect objects in RGB images, projecting the image mask of each detection to the 3D scene using the associated depth information. Then, our proposal models each object in the workspace with a 3D bounding box, which captures the essential geometric information of the real object (pose and size). Each model is anchored to its semantics i.e., a confidence score about the categories it belongs to (e.g., chair, microwave, tv, etc.) and a set of flags indicating the visibility of the vertices of its bounding box that has been visible. The latter affords us to address the previously described instance duplication problem since this estimation provides useful insights on whether an object is expected to be larger than detected due to partial views, occlusions, etc. We exploit this information and new detections to refine the geometric knowledge of the objects in the map by sharpening their pose and size, even merging two object instances if they are considered to belong to the same physical object. In addition, to ensure the consistency of the resulting map over time against dynamic objects, we take into consideration not only detections but also non-detections of previously mapped objects. The non-detection concept refers to the missing detection of an object that was expected to be re-observed at a certain location. This concept is defined from a characterization of the object detector response, and permits us to decrease the confidence of the non-detected objects in the map, resulting in their removal after multiple non-detections.

In order to evaluate the performance of *LTC-Mapping*, we carried out a set of experiments in different indoor environments with representative settings from the Robot@VirtualHome ecosystem [14]. Moreover, to test the adaptability to dynamic environments, we include experiments where objects appear and disappear from those environments. From the obtained results, we demonstrate the suitability of *LTC-Mapping*, which also exhibits a superior performance than a state-of-the-art alternative [13], achieving approximately a 70% of object classification accuracy while keeping a low error in modeling their geometry. The method implementation is publicly available at https://github.com/MAPIRlab/LTC-Mapping (accessed on 12 July 2022).

## 2. Related Work

In the context of mobile robots working in human-centered environments, the geometric modeling of the environment has been widely addressed and has reached a considerable degree of maturity [15,16]. A purely geometric map is sufficient for a robot carrying out low-level tasks (e.g., navigation), but not for reasoning and interacting with the workspace and its elements. In the last decades, leveraging the advances in deep learning for object detection, multiple contributions have been proposed to enrich geometric maps with semantic information, enabling a high-level understanding of the environment. The latter is known in the literature as the semantic mapping problem [1]. Next, we review the most relevant works of semantic mapping and object detection. For an exhaustive review of these research areas, the interested reader can refer to [17,18].

### 2.1. Semantic Mapping

Semantic mapping refers to the problem of building and maintaining a representation of the spatial elements present in the environment, linking both their geometric and semantic information [1,3,19]. However, according to how the semantic information is represented in the map, the approaches to semantic mapping can be divided into two groups: dense and object-oriented.

On the one hand, dense semantic mapping contributions annotate spatial representations (e.g., 2D cells or 3D voxels) with object information acquired from images, but they do not consider objects as instances. For example, Regier et al. [20] employ a Convolutional Neural Network (CNN) for object detection to label cells of a 2D grid map with semantic information, aiming to exploit it for obstacle avoidance. Moving from 2D to 3D, Tateno et al. [21] and McCormac et al. [22] exploit real-time SLAM to incrementally build 3D surfel-based representations, where each surfel is annotated with an object class and a confidence score. Xiang et al. [23] propose a Data Associated Recurrent Neural Network (DA-RNN) to joint mapping and semantic labeling of a 3D scene through voxels from RGB-D videos. Other works follow the same approach, i.e., labeling 3D global representations, but employing different representation models such as Li et al. [24] and Sun et al. [25], which employ octree maps in order to reduce the error from pose estimation.

On the other hand, object-oriented methods, which represent objects in the map as individual instances anchored with semantic information, exhibit some advantages that justify their extensive applicability (e.g., a more compact map representation, a more straightforward exploitation, etc.). For example, Dengler et al. [13] present an online semantic mapping method that represents instances of objects as 2D polygons over the geometric map, which are annotated with semantic information (i.e., object class and a likelihood). The available information in the semantic map is updated over time to provide robustness against dynamic changes in the scene and to avoid instance duplication. In contrast, Sünderhauf et al. [11] propose to extend the geometric representation of objects to 3D by using point clouds but considering the world as static, hence objects are just updated when they are detected. Similarly, Narita et al. [26] and Grinvald et al. [27] propose volumetric semantic mapping methods based on voxels, which are labeled with semantic information coming from object detection CNNs integrated over time. Since voxels are computationally demanding, Nakajima et al. [28] propose to annotate the semantic information to 3D regions rather than each element of the scene (e.g., surfels and voxels), which significantly alleviate the computational cost. More compact approaches are presented by Deeken et al. [29] and Wang et al. [30], which uses 3D bounding boxes to represent objects, storing just the essential geometric information (i.e., size and pose). However, a common drawback of these works is that they do not consider instance duplication and dynamic objects challenges simultaneously or even consider either of them, thus compromising the long-term consistency of semantic maps.

*LTC-Mapping* is framed within the object-oriented semantic mapping philosophy, but distances from previous works in: (i)the explicit tackling of the instance duplication problem, producing just a representation in the map for each physical object in the real-world (one-to-one mapping), and (ii) the consideration of the world as dynamic, so the map is not only updated with new object detections, but also with missing detections of objects already represented in the map. These features aim to provide a reliable semantic map useful in the long-term robot operation.

### 2.2. Object Detection and Semantic Segmentation

To populate a map with high-level information from the objects in the environment, it is crucial to dispose of a trustworthy object detector. Initially, approaches based on machine learning techniques exhibited successful results in this area. A common pipeline of those works is the extraction of image descriptors such as Scale-Invariant Feature Transform (SIFT) or Speeded-Up Robust Features (SURF) and their classification through machine learning algorithms, for example Supported Vector Machines (SVMs) in Pontil et al. [31] or Bag-of-Words in Nister et al. [32]. However, a serious drawback of these works comes when different object categories can exhibit similar features, hence the results tend to be ambiguous. The latter is tackled in posterior works by including contextual information in the classification. In this sense, a widely exploited contextual information is the object–object relations, e.g., chairs are usually found close to tables. For example, Valentin et al. [33] classify the faces of a mesh representing an object in the scene by considering its relations with near objects through a Conditional Random Field (CRF). Extensions to this work are proposed by Ruiz-Sarmiento et al. [34,35,36], where CRFs are used along with ontologies [37] to increase the efficiency of object detection.

More recently, deep learning neural networks are showing a substantial maturity, outperforming machine learning-based methods [38]. Among deep learning-based methods, we highlight popular networks which outputs bounding boxes of objects in the image annotated with the object class and a confidence score, such as YOLO [39], Single-Shot Detector (SSD) [40], and RetinaNet [41]. However, by using these kinds of networks, it becomes necessary to post-process the bounding box to remove the background of the object. Going a step further, semantic segmentation networks also provide a mask of the pixels belonging to the object. The latter is a key aspect for obtaining accurate representations of objects and hence semantic segmentation networks are the most widely used for semantic mapping. The most popular semantic segmentation network is Mask R-CNN [42], but also others such as SceneCut [43], RefineNet [44], and Mask${}^{X}$ R-CNN [45] are widely used.

## 3. *LTC-Mapping *

**Figure 2:**
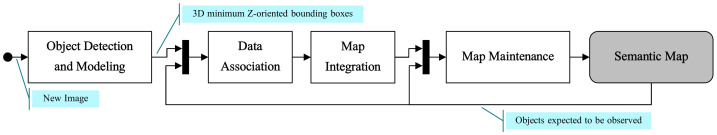
Pipeline of *LTC-Mapping*. White boxes represent data processing stages while gray box stands for the formal representation of the semantic map, which contains the prior knowledge.

Our proposal aims to incrementally build and maintain a semantic map of a dynamic environment from images captured by an RGB-D camera mounted on a mobile robot. To do so, for each input image, four stages are carried out: (i) object detection and modeling, where objects are identified and characterized from input images, (ii) data association, which enables determining the correspondences between the new detected objects and the objects already represented in the map, (iii) map integration, to refine and extend the knowledge available in the map, and (iv) map maintenance, to keep the map reliable and up-to-date. The complete method pipeline can be seen in Figure 2.

It must be noticed that, since *LTC-Mapping* works on an image-by-image basis, we require knowing at each time instant the camera pose w.r.t. the world frame ($T_{C}^{W}$), in order to fuse the new gathered information into a global semantic map. However, because the camera is normally fixed to the robot, the camera pose w.r.t. the robot ($T_{C}^{R}$) can be assumed to be known. Therefore, knowing that $T_{C}^{W}=T_{R}^{W}\;T_{C}^{R}$, the problem is reduced to obtain the robot-world relative pose ($T_{R}^{W}$). The latter is retrieved in this work through the well-known Adaptive Monte Carlo Localization (AMCL) method implemented in the AMCL ROS package (http://wiki.ros.org/amcl) (accessed on 12 July 2022). Hence, from now on, we consider that the camera pose is known. This way, an object-based semantic map is composed of a 2D geometric map that works as a common (world) reference system, and a set of representations of objects $O^{M}=\left\{o_{1},\cdots ,o_{n}\right\}$ modeling the objects found in the robot workspace. Next, we describe in further detail the four core stages of our proposal.

### 3.1. Object Detection and Modeling

A trustworthy detection of the objects in the robot workspace is a keystone task in the generation of object-based semantic maps, CNN-based techniques being the de-facto choice in modern solutions. Of special interest in mobile robotics applications are those CNNs that, in addition to the bounding boxes of each detected object in the image, their category *c*, and associated confidence scores *s*, also provide a mask over the pixels in the image belonging to said objects. When using an RGB-D camera, this permits the mapping of those masks from the RGB to the Depth image in order to retrieve their geometry in the scene. This work considers the utilization of this type of networks.

However, masks provided by this kind of CNNs are not error-free and tend to include pixels belonging to adjacent elements in the image. The latter becomes a major concern in order to obtain a 3D representation of the object. To address this problem, the pixel mask is pre-processed by applying the thinning morphological operation [46], removing possible outliers at the object boundaries while keeping the topological skeleton of the object (see Figure 3.1).

In order to obtain a 3D representation of the objects, we first project the pixels of the mask into the 3D space w.r.t. the camera frame to obtain a local point cloud $P^{C}=\left[X^{C},Y^{C},Z^{C}\right]$ as follows:

(1) $$\left[\begin{array}{c} X^{C}\\ Y^{C}\\ Z^{C} \end{array}\right]=Z^{C}K^{-1}\left[\begin{array}{c} x^{\prime }\\ y^{\prime }\\ 1 \end{array}\right]=Z^{C}\left[\begin{array}{ccc} \frac{1}{f} & 0 & \frac{-x_{0}}{f}\\ 0 & \frac{1}{f} & \frac{-x_{0}}{f}\\ 0 & 0 & 1 \end{array}\right]\left[\begin{array}{c} x^{\prime }\\ y^{\prime }\\ 1 \end{array}\right]=Z^{C}\left[\begin{array}{c} \frac{x^{\prime }-x_{0}}{f}\\ \frac{y^{\prime }-y_{0}}{f}\\ 1 \end{array}\right]$$

where *K* is the matrix containing the camera intrinsic parameters (focal length of the camera *f*, and camera center ($x_{0},y_{0}$)), while ($x^{\prime },y^{\prime }$) refers to the coordinates of the pixels in the mask and $Z^{C}$ to their depth.

A common drawback in the estimation of the 3D representation occurs when the detected object includes holes (e.g., a chair with bars in the back). This implies that the resulting point cloud $P^{C}$ also contains outliers that are not part of the object itself. Hence, to filter out these outliers, our method applies a spatial density-based clustering (DBSCAN [47]), producing the result illustrated in Figure 3.3. The inliers are considered as part of the object and hence are transformed from the camera frame to the world frame, obtaining the object point cloud $P^{W}$.

Finally, to conclude the pipeline of *LTC-Mapping* to represent objects’ geometry, we propose the use of 3D minimum oriented bounding boxes, that is, the boxes that enclose the minimum volume of each object. As discussed, this object modeling exhibits low memory requirements while preserving the essential geometric information of objects (i.e., pose and size). Since objects in the real-world are usually resting on surfaces parallel to the ground, we make the assumption that they can only appear rotated in the vertical axis, hence the bounding box is reduced to Z-oriented. Based on this fact, for each object, we obtain its representation by computing the best-fitting 3D bounding box $B=[c_{x},c_{y},c_{z},\theta ,w,h,d]$ of $P^{W}$ (see Figure 3.4), which is defined by its pose (position of the centroid $\left[c_{x},c_{y},c_{z}\right]$ and orientation θ) and size (width *w*, height *h*, and depth *d*).

Next, we enrich these bounding boxes with semantic information: a confidence vector of the possible object classes the objects could belong to, and an estimation about the visibility of the bounding boxes vertices in the images. The former provides per-class confidence scores $S=[s_{0},\cdots ,s_{n}]$ with n=r+1, where *r* is the number of object categories recognizable by the used CNN. Notice that we also include an additional class, *Other*, to account for the possibility of the object being misdetected. As for the estimation of the observed vertices, we build the vector $V=[b_{0},\cdots ,b_{7}]$ of the bounding box B, where each $b_{i}$ represents a *Visible* flag, stating if the vertex was visible in the observation or not. This estimation is carried out by re-projecting each vertex of the bounding box into the image plane and checking if they are within the camera field-of-view or if they are occluded. Occlusions are checked by applying Z-buffering techniques [48] over the re-projected vertices, i.e., comparing the depth of the re-projected vertex with the value of the corresponding pixel in the depth image. The result of this step is shown in Figure 3.5. From this information, we can infer which sides of the object were occluded in the observation, which is taken into account in the next stage of *LTC-Mapping*: data association. This way, each object $o_{i}\in O^{M}$ is represented in the semantic map by the 3-tuple $o_{i}=\left(B,S,V\right)$. For the sake of simplicity, we refer to this object representation as the object itself, where it does not create confusion.

### 3.2. Data Association

The proposed data association follows an image-to-model approach. In this process, the objects detected in an image captured at time *t*, forming the set $O_{t}^{I}$, are matched against the already instantiated objects in the map that must be visible from the current camera point of view, represented by ${\hat{O}}_{t-1}^{M}$. This restriction aims to maintain spatial coherence and reduce the number of mismatches. Thus, if a new detected object matches with a previously instantiated one, we integrate their information. Otherwise, the object representation is initialized in the semantic map.

To determine whether or not a detected object $o_{i}\in O_{t}^{I}$ refers to an already existing object ${\hat{o}}_{j}\in {\hat{O}}_{t-1}^{M}$, we measure the similarity between their respective bounding boxes $B_{i}$ and $B_{j}$. The latter is performed by computing the average Euclidean distance between pairs of nearest vertices from both bounding boxes. The choice of this metric is motivated by the fact that it is able to compare the pose and scale of two bounding boxes at once. It is computed as follows:

(2) $$similarity\left(B_{i},B_{j}\right)=\frac{1}{8}\sum_{k=0}^{7}\sqrt{{\left(p_{i,k}-p_{j,k}\right)}^{2}}.$$

where $p_{i,k}$ and $p_{j,k}$ refer to the *k*-th bounding box vertex of $o_{i}$ and ${\hat{o}}_{j}$, respectively.

It is important to note that this metric is not fair when comparing two representations referring to the same real object but with different observability conditions, i.e., one comes from a complete observation while the second comes from a partial observation. Thus, considering the visibility conditions given by the previously mentioned *Visible* flag of each corner, we discern two different scenarios, adapting the metric accordingly:

- **Three or more pairs of vertices are visible**. A pair of vertices is considered as visible as long as each vertex is *Visible* in their respective bounding box. Based on this knowledge, we can assume that at least two dimensions of their size are known and are not expected to be highly modified with new detections. Therefore, the distance between these vertices is more informative than the distance between non-visible vertices, which may not represent a real vertex of the object. Hence, under this scenario, the metric is particularized just to the average distance between pairs of non-occluded vertices.
- **Less than three pairs of vertices are visible**. In this case, given the lack of reliable information, we compute the average distance between all pairs of vertices.

Hence, an object $o_{i}$ is matched with an instantiated object ${\hat{o}}_{j}$ as long as the similarity function returns a value lower than a given threshold $\tau_{max}$. Note that, in this work, we consider that an already instantiated object can match with two different new object detections since state-of-the-art object detectors tend to over-segment objects in the image [27]. Thus, a global optimization step in the data association process is not required.

**Figure 4:**
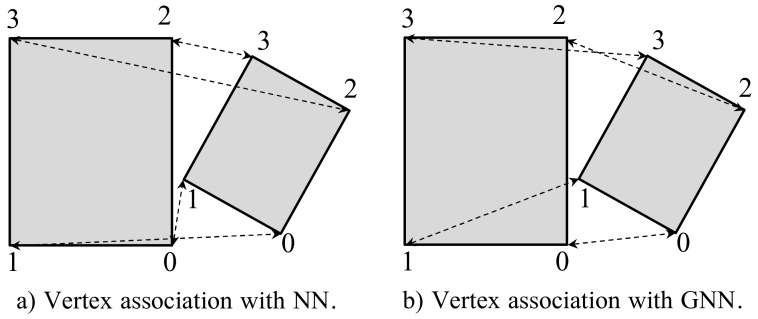
Example of the difference between the association using NN and GNN. On the left, the result using NN, being a local minimum where the vertices are mismatched. On the right, the results obtained using GNN, where the vertices are correctly matched to obtain a global minimum of distances between vertices.

It can be noticed that measuring the distance between pairs of vertices requires knowledge about the correspondences between said pairs. This task is usually solved in similar problems by employing Nearest Neighbor (NN) techniques. In this context, NN works as follows: for each vertex of $B_{i}$, NN associates it with its nearest vertex from $B_{j}$. However, it computes the optimal association pair one-by-one, hence the global result could not be the optimal one as it can be seen in Figure 4a. A well-known solution to this problem is using Global Nearest Neighbor (GNN), which yields the global result by minimizing the distance between vertices globally rather than one-by-one (see Figure 4b). In our case, this algorithm would become highly time-consuming: it requires computing $8^{8}$ possible vertices’ combinations. Doing this for each possible objects’ association could compromise the online execution of the semantic mapping method.

However, by particularizing GNN to our problem and including prior knowledge, the computation time can be significantly reduced. Concretely, since we assume that bounding boxes lay on surfaces parallel to the ground (i.e., *x* and *y*-axis rotations are null), both top and bottom vertices are equal except for the *z*-coordinate. The latter leads to reducing the task just to associate the vertices of one of the two faces (i.e., top or bottom), hence the possible combinations are $4^{4}$. Moreover, considering that the relative position between the vertices of one face of the bounding box is known, this number is reduced to four valid combinations. This particularization of GNN enables the online execution of the proposed method.

### 3.3. Map Integration

Once the matching between new objects’ detections $O_{t}^{I}$ and those already instantiated in the map $O_{t-1}^{M}$ is completed, we proceed to integrate this information in order to expand and refine the semantic map, obtaining $O_{t}^{M}$. To do so, we leverage the reliability of the information by considering the observability conditions of objects given by the *Visible* flags. Exploiting the flags, we consider that the bounding box of an object is *Defined* if the size of the three dimensions is known. The latter leads to suppose that the object geometry, once represented in the map, is not expected to be highly modified with new observations.

Thus, depending on whether the bounding box of the new detected object $B_{i}$ and the bounding box of its matched object on the map $B_{j}$ are or are not defined, we consider four possible scenarios:

- **$B_{i}$*****Defined*****and $B_{j}$*****Defined***. In this scenario, both bounding boxes should cover the entire object and are expected to be similar. Hence, the resulting bounding box comes from separately averaging both size and orientation. The resulting bounding box remains as *Defined*.
- **$B_{i}$ not*****Defined*****and $B_{j}$*****Defined***. In this case, the new observation is partial (e.g., due to occlusions), so the obtained bounding box does not completely represent the real object. Therefore, we do not modify the bounding box of the already instantiated object.
- **$B_{i}$*****Defined*****and $B_{j}$ not*****Defined***. Similarly to the above case, the bounding box of the new detection being the most complete one, we replace the instantiated bounding box $B_{j}$ with the new one $B_{i}$, as it should represent the real object more accurately. Hence, the bounding box in the map is now set as *Defined*.
- **$B_{i}$ not*****Defined*****and $B_{j}$ not*****Defined***. Under this scenario, both bounding boxes belong to partial observations. Hence, in this case, we compute the minimum bounding box that encloses both $B_{i}$ and $B_{j}$. However, this operation is highly sensitive to the orientation of both bounding boxes and, consequently, minor errors in orientation affect the bounding box size highly. This leads to the fact that, after integrating multiple partial observations, the size tends to become larger and larger. To overcome this problem, we apply to the resulting bounding box a slight 3D morphological erosion [49], which prevents an endless expansion until a *Defined* detection is received.

Referring to the semantic knowledge anchored to each object representation in the map, we update and normalize the confidence value of each object class in the accumulated confidence vector (s∈S) of the instantiated object ${\hat{o}}_{j}$ as follows:

(3) $$s_{j,c}=\frac{s_{i,c}+D\;s_{j,c}}{\sum_{l=0}^{r}s_{i,l}+D\;s_{j,l}},c=0,\cdots ,n.$$

where *D* is the number of observations of the object represented in the map and *n* is the number of known classes by the neural network plus the class Other.

### 3.4. Map Maintenance

Building on the statement of a dynamic environment, it is required not only to update the map with new detections, but also to consider those objects that were expected to appear in the current image but are missing. In this work, we incorporate the concept of non-detection to address this fact. The 3-tuple $o_{nd}$ of a non-detection is modeled as:

- $B_{nd}$: a bounding box with no size located at the position where the instantiated object was expected to be found.
- $S_{nd}$: the confidence value of the class *Other* is set with a value $v_{Other}$ obtained from a characterization of the object detector response (i.e., true positives, false negatives, etc.) obtained from a previous analysis. The remaining classes are set uniformly as:
  (4) $$s_{c}=\frac{1-v_{Other}}{r},\;\;\;c=0,\cdots ,r.$$
  where *r* is the number of object classes detectable by the neural network.
- $V_{nd}$: an empty visibility vector.

Thus, considering the objects already instantiated in the map that fall within the camera field of view and are not occluded by other instantiated objects, if they did not match a new object detection, we associate them with a non-detection. The effect of integrating a non-detection is a decrease of the confidence that the model represents a real object and an increase of the confidence of the *Other* class. In this sense, objects whose most representative class is *Other* are more likely to represent an object that is no longer in the environment or produced by a false positive, therefore being removed from the semantic map. As a consequence, objects obtaining multiple non-detections are prone to disappear from the map.

## 4. Experimental Setup

In order to evaluate the performance of *LTC-Mapping*, we have carried out a number of experiments comparing its outcome with the one from a state-of-the-art approach [13]. The experiments have taken place in different representative environments from the Robot@VirtualHome ecosystem [14]. A further description of both the dataset and the setup employed in the experiments is provided in Section 4.1. The implementation details of the evaluated methods are explained in Section 4.2. Then, the obtained results are reported in Section 5.

### 4.1. Robot@VirtualHome Ecosystem

**Figure 5:**
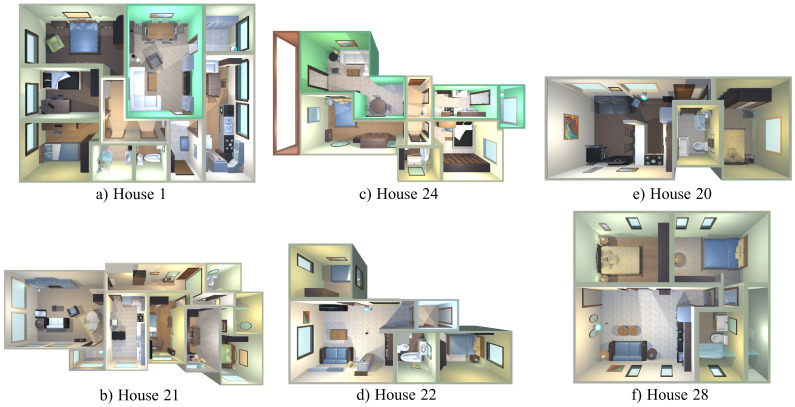
Environments from Robot@VirtualHome ecosystem used in the experimental validation of the method. Large, medium, and small size environments are depicted in (**a**,**b**), (**c**,**d**) and (**e**,**f**), respectively.

Robot@VirtualHome [14] is a publicly available ecosystem (https://github.com/DavidFernandezChaves/RobotAtVirtualHome) (accessed on 12 July 2022) composed by a set of 30 virtual realistic-looking environments recreated from real households, containing objects belonging to 60 different object types. The ecosystem includes a virtualization of the Giraff robot [50], which is equipped with a 2D laser scan and an RGB-D camera mounted at a height of 1.05 m from the floor. For evaluation purposes, we have selected a set of six representative environments, which are shown in Figure 5: House 1 and House 21 as large size environments, House 22 and House 24 as normal size, and House 20 and House 28 as small size scenarios containing objects located close to each other. For the sake of reproducibility, the navigation paths followed by the robot are those provided by the dataset under the Wanderer robot behavior. In the experiments carried out, these paths are followed twice in a row. In order to simulate a dynamic environment, the location of certain object was modified in the second lap.

### 4.2. Implementation Details

In order to detect objects in the scene, and for both methods under consideration, we make use of a state-of-the-art object detection CNN. Namely, we use the tool Detectron2 [51], which incorporates an implementation of Mask R-CNN [42], pre-trained on the Microsoft COCO dataset [52]. This network is able to detect 80 different object classes, but just 26 are relevant to this work, since the remaining ones are categories scarcely appearing in the considered environments, e.g., animals (giraffe, elephant, etc.) and vehicles (bus, train, etc.), etc.

To implement the proposed method, we have leveraged our recent work ViMantic [53], a distributed robotic architecture for semantic mapping. This tool includes, among other features, a formal model for the definition and managing of the semantics of the environment, distributed execution capabilities through a client–server design, user interfaces for the visualization and interaction with the maps, and public availability. In the experiments carried out, a robot—operating as a client—has been instantiated in the considered environments, which process the acquired information (i.e., RGB-D images) using an ROS node in charge of detecting and modeling the objects. The obtained objects are sent to the server side of ViMantic, which performs the data association, map integration, and map maintenance stages (recall Section 3). In order to match two object detections, we set the $\tau_{max}$ parameter to 1 m. (recall Section 3.2). In the case of the method proposed by Dengler et al. [13], its implementation is available as a ROS package, hence the complete algorithm is implemented on the client side, so the server is used just for visualization.

As for hardware specifications, the server side ran on a computer with an Intel Core i7-5700HQ CPU at 2.70 GHz, 16 GB DDR3 memory RAM at 800 MHz, and an Nvidia GeForce GTX 960M GPU with 2 GB of memory. In contrast, as the client requires higher computational resources, we employed a computer with an Intel Core i7-8750H CPU at 2.20 GHz, 16 GB DDR4 memory RAM at 1333 MHz and an Nvidia GeForce GTX 1070 GPU with 8 GB.

## 5. Experimental Results

This section reports the obtained results of the experiments carried out with regard to different aspects worth discussing. First, Section 5.1 provides quantitative results regarding the geometric and semantic modeling of objects. Then, Section 5.2 yields some examples of semantic maps to qualitatively check the performance of the considered methods. Finally, Section 5.3 reports on the computational time required by the different stages of *LTC-Mapping*.

### 5.1. Quantitative Results

We first discuss how accurate our method is for modeling both the geometry and semantics of the objects in the environment. Regarding objects’ geometry, Table 1 illustrates the yielded results in this regard. Since the method from [13] employs 2D bounding boxes to represent objects in the map, we use the Intersection over Union (IoU) function to measure its performance. Note that *LTC-Mapping* uses 3D bounding boxes, so, for a fair comparison, we project them onto the XY-plane. The results show a superior performance of *LTC-Mapping*, achieving on average a ∼0.092 higher IoU value. Additionally, we assess the precision of the 3D models obtained by our proposal by measuring the Volumetric Intersection over Union (VIoU). Note that the intersection between two 3D oriented bounding boxes is not a 3D bounding box, hence computing the VIoU is not straightforward. Thus, instead, we employ an approximation of the VIoU applying the Pick’s theorem [54].

Although in this case a comparison with the method from [13] is not possible, having notions of the meaning of this metric, it can be said that *LTC-Mapping* is able to properly represent the three dimensions of the objects. It is important to mention that, when computing IoU and VIoU metrics, the complete ground-truth of each object is considered. However, instantiated objects in the map could have not been observed completely, their obtained representations being partial. This fact prevents both metrics to reach higher values. This is also noticeable in the position error, which measures the distance between the centers of mass of the ground-truth and the evaluated object. Nevertheless, this error is considerably reduced by our proposal when compared to its counterpart, improving it by 34.5 cm on average. These metrics could be further reduced by introducing active perception techniques motivating the inspection of partially observed objects in the map, situations that can be easily detected in our method by employing the visibility flags V.

Referring to the semantic information available in the map, we compare the top-1 object class (i.e., the class with maximum score according to the confidence score vector S) of each represented object with the associated semantic label of the ground-truth. The results depicted in Table 2 demonstrate that *LTC-Mapping* is able to build reliable semantic maps, keeping a low number of false detections in comparison with [13]. This proves that the proposed non-detection concept is beneficial to maintain a true representation of the real-world over time. Moreover, for most of the environments, our method outperforms both in precision and recall to the state-of-the-art method. However, note how the number of false negatives is not insignificant for either semantic mapping method. The latter is explained by the fact that the robot followed predefined inspection paths in which not all objects were visible. That is, all the objects in the different houses were considered for this metric computation, not just those observed by the robot. If needed, this metric could be improved by using exploration algorithms maximizing the area covered by the robot [9].

Looking at Table 1 and Table 2 and comparing the results between the first and the second lap, it can be noticed that both evaluated methods are able to adapt properly to dynamic environments, showing *LTC-Mapping* a slight improvement in comparison with Dengler et al. [13]. In this context, both methods maintain the accuracy and recall of the semantic map over time, which means that the map remains useful after changes in the environment. Furthermore, the geometric information is refined, reducing the error in position while increasing the IoU and VIoU values.

### 5.2. Qualitative Results

**Figure 6:**
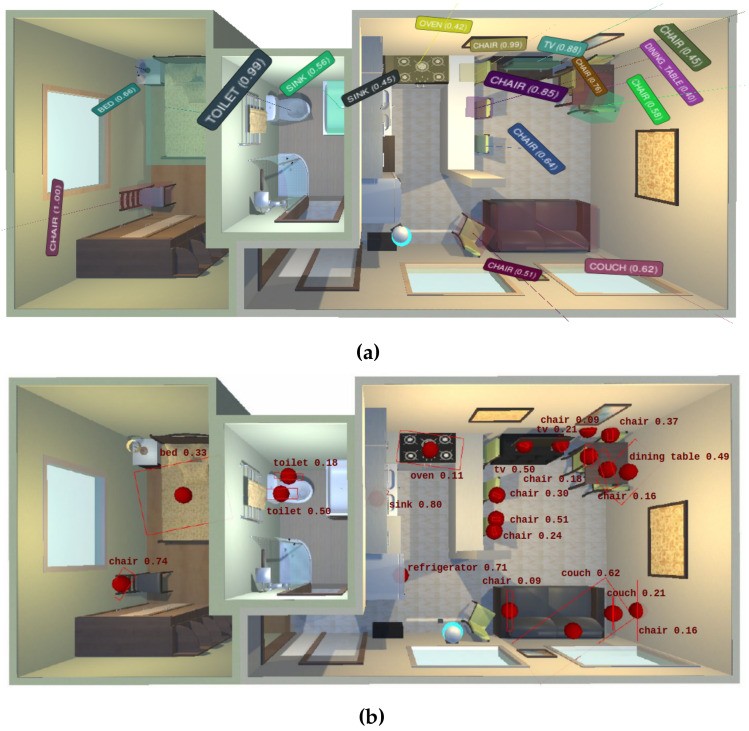
Semantic maps built by each evaluated method in the environment House 20 from the Robot@VirtualHome ecosystem. (**a**) *LTC-Mapping*; (**b**) Dengler et al. [13].

In order to qualitatively evaluate the considered methods, an instance of the semantic map built by each one, layered over the ground-truth information, is shown in Figure 6. Visually, two relevant aspects can be highlighted where our method outperforms the results from [13]: (i) the accuracy and completeness in the geometric representation of objects, including essential 3D information, and (ii) the unique representation in the map of each perceived physical object, thus successfully dealing with the instance duplication problem.

### 5.3. Analysis of Computational Time

This section aims to validate the online operation of *LTC-Mapping*. For this purpose, we have designed a more restrictive scenario than the one used in the previous experiments, where a computer running the server side of *ViMantic* is not available, that is, both server and client must run on the same computer (presumably the robot onboard one). This way, for running the experiments described in this section, we have used a laptop equipped with an Intel Core i7-8750H CPU at 2.20 GHz, 16 GB DDR4 memory RAM at 1333 MHz, and an Nvidia GeForce GTX 1050M GPU with 4 GB of memory.

Table 3 summarizes the average computational times required by the key stages of our method. The obtained results show that our method is able to work online at ∼2 Hz even in this more restrictive scenario, as long as the object detection stage is executed in parallel. In robots with constrained resources, the time consumed by the detection of objects could be reduced by downsizing the input images (at the cost of not detecting far objects), or by replacing the object detection network with a more lightweight one (e.g., YOLO [39], at the cost of accuracy).

## 6. Conclusions and Future Work

This work contributes a novel object-based semantic mapping method, coined *LTC-Mapping*, which continuously adapts to changes in the environment, extending the usefulness of the produced maps in long-term robot operation. Object-based maps are incrementally built from a sequence of RGB-D images, fusing partial information from objects into a global representation. In this sense, for each image, we extract objects and model them using 3D bounding boxes, whose vertices are annotated with information about their visibility in the given image. The resulting bounding boxes are enriched with semantic information (i.e., confidence scores about their belonging classes) and are integrated in the global model through a data association stage. In this stage, exploiting the information about the visibility of the bounding boxes’ vertices, we refine and extend the knowledge available in the semantic map, hence palliating the instance duplication issue. Moreover, we introduce the concept of non-detection, which refers to the missed detection of previously detected objects in the location where they should appear. The latter is exploited to keep the map up-to-date by removing objects with multiple consecutive non-detections, which indicates that the object has been moved.

The performance of the proposal has been validated with a set of experiments carried out in different environments of the Robot@VirtualHome ecosystem. In these experiments, our method reported a superior performance than a state-of-the-art alternative regarding IoU, position error, and F1-score metrics. Examples of built semantic maps have also been depicted, which permits to visually check how *LTC-Mapping* successfully handles the instance duplication issue. Additionally, its online operation has also been validated, reporting for the experiments a working frequency of ∼2 Hz.

For future work, we plan to incorporate not only objects to the semantic map, but also structural elements of the scene (e.g., walls), and to exploit them and the contextual information that they provide to refine the map. We also plan to explore the utilization of active perception methods for the further inspection of partially visible objects. Additionally, we also consider deploying an extended version of *LTC-Mapping* including the aforementioned thoughts in the real-world under a multi-robot scenario.

## Figures and Tables

**Figure 3:**
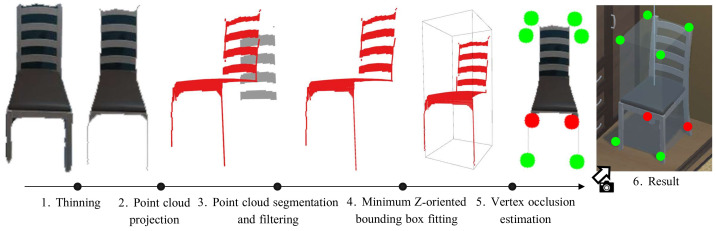
Overview of the process used to build the models of the detected objects. Each detected object mask is first subjected to a thinning step to remove spurious pixels while maintaining the object structure. Then, it is projected in 3D space to obtain a point cloud which we segment and filter—removing the gray points shown. From the resulting point cloud, we fit a Z-oriented bounding box, whose vertices we evaluate to check if they are visible. The latter is represented in the figure using red dots for the non-visible vertices and green dots for the visible ones.

**Table 1:** Geometric evaluation for each environment of the evaluated methods through the Intersection over Union (IoU) function and its Volumetric form (VIoU), and the error between the centers of masses of the ground-truth and the object representation in the map. Best results are marked in bold.

		Lap 1			Lap 2		
Environment	Method	IoU	VIoU	Position Error (m)	IoU	VIoU	Position Error (m)
House1	*LTC-Mapping *	**0.478**	0.377	**0.255**	**0.365**	0.268	**0.247**
	Dengler et al. [13]	0.232	–	0.564	0.212	–	0.445
House20	*LTC-Mapping *	**0.472**	0.312	**0.222**	**0.488**	0.347	**0.203**
	Dengler et al. [13]	0.307	–	0.563	0.461	–	0.501
House21	*LTC-Mapping *	**0.396**	0.196	**0.302**	**0.397**	0.299	**0.268**
	Dengler et al. [13]	0.390	–	0.597	0.356	–	0.586
House22	*LTC-Mapping *	**0.465**	0.352	**0.151**	**0.476**	0.313	**0.153**
	Dengler et al. [13]	0.294	–	0.543	0.259	–	0.579
House24	*LTC-Mapping *	**0.392**	0.227	**0.306**	0.405	0.246	**0.264**
	Dengler et al. [13]	0.319	–	0.842	**0.454**	–	0.515
House28	*LTC-Mapping *	**0.396**	0.264	**0.199**	**0.487**	0.285	**0.220**
	Dengler et al. [13]	0.365	–	0.626	0.463	–	0.569
Average	*LTC-Mapping *	**0.433**	0.288	**0.239**	**0.436**	0.293	**0.226**
	Dengler et al. [13]	0.318	–	0.622	0.368	–	0.532

**Table 2:** Evaluation of the semantic information available in the maps built by each evaluated method for each environment. The metrics employed refer to the number of true positives (TP), false positives (FP), false negatives (FN), accuracy, recall, and F1-score. Note that, for this evaluation, we compare the top-1 class of each object represented in the map with the label of the ground-truth. Best results are marked in bold.

		Lap 1						Lap 2					
Environment	Method	TP	FP	FN	Accuracy	Recall	F1-Score	TP	FP	FN	Accuracy	Recall	F1-Score
House1	*LTC-Mapping *	**15**	**4**	**27**	**78.95%**	**35.71%**	**49.18%**	**16**	**4**	**26**	**80.00%**	**38.10%**	**46.38%**
	Dengler et al. [13]	5	38	37	11.63%	11.90%	11.76%	6	27	36	18.18%	14.29%	16.00%
House20	*LTC-Mapping *	**10**	**3**	**9**	**76.92%**	**52.63%**	**62.50%**	**11**	**4**	**8**	**73.33%**	**57.89%**	**64.71%**
	Dengler et al. [13]	6	19	13	24.00%	31.58%	27.27%	7	15	12	31.82%	36.84%	34.15%
House21	*LTC-Mapping *	**19**	**4**	**25**	**82.61%**	**43.18%**	**56.72%**	**18**	**5**	**26**	**78.26%**	**40.91%**	**52.94%**
	Dengler et al. [13]	7	33	37	17.50%	15.91%	16.67%	7	41	37	14.58%	15.91%	15.22%
House22	*LTC-Mapping *	**6**	**2**	**6**	**75.00%**	**50.00%**	**60.00%**	**6**	**3**	**6**	**66.67%**	**50.00%**	**57.14%**
	Dengler et al. [13]	**6**	11	**6**	35.29%	**50.00%**	41.38%	**6**	15	**6**	28.57%	**50.00%**	36.36%
House24	*LTC-Mapping *	11	**6**	13	**64.71%**	45.83%	**53.66%**	**10**	**4**	**14**	**71.43%**	**41.67%**	**52.63%**
	Dengler et al. [13]	**12**	32	**12**	27.27%	**50.00%**	35.29%	7	23	17	23.33%	29.17%	25.93%
House28	*LTC-Mapping *	**4**	**7**	**17**	**36.36%**	**19.05%**	**25.00%**	**6**	**7**	**15**	**46.15%**	**28.57%**	**33.33%**
	Dengler et al. [13]	**4**	19	**17**	17.39%	**19.05%**	18.18%	4	20	17	16.67%	19.05%	17.78%
Average	*LTC-Mapping *	**10.83**	**4.33**	**16.17**	**69.09%**	**41.07%**	**51.18%**	**11.17**	**4.50**	**15.83**	**69.31%**	**42.86%**	**51.19%**
	Dengler et al. [13]	6.67	25.33	20.33	22.18%	29.74%	25.09%	6.17	23.50	20.83	22.19%	27.54%	24.24%

**Table 3:** Average computation time per frame for each of the key stages of *LTC-Mapping* with RGB-D input images of 640 × 480 resolution. Note that the object detection is computed in parallel to our pipeline and hence is not included in the total time of *LTC-Mapping*.

Stage	Avg. Time (ms)
Object Modeling	137.58
Data Association	141.91
Map Integration	143.05
Map Maintenance	76.27
Total	498.81
Object Detection (Detectron2)	494.84

## Data Availability

Not applicable.
